# An Interactive Image Segmentation Method Based on Multi-Level Semantic Fusion

**DOI:** 10.3390/s23146394

**Published:** 2023-07-14

**Authors:** Ruirui Zou, Qinghui Wang, Falin Wen, Yang Chen, Jiale Liu, Shaoyi Du, Chengzhi Yuan

**Affiliations:** 1 School of Physics and Mechanical and Electrical Engineering, Longyan University, Longyan 364012, China; 2 School of Software Engineering, Xi’an Jiaotong University, Xi’an 710049, China; 3 Institute of Artificial Intelligence and Robotics, Xi’an Jiaotong University, Xi’an 710049, China; 4 Department of Mechanical, Industrial and Systems Engineering, University of Rhode Island, Kingston, RI 02881, USA

**Keywords:** interactive image segmentation, attention, cross-stage feature aggregation, model complexity

## Abstract

Understanding and analyzing 2D/3D sensor data is crucial for a wide range of machine learning-based applications, including object detection, scene segmentation, and salient object detection. In this context, interactive object segmentation is a vital task in image editing and medical diagnosis, involving the accurate separation of the target object from its background based on user annotation information. However, existing interactive object segmentation methods struggle to effectively leverage such information to guide object-segmentation models. To address these challenges, this paper proposes an interactive image-segmentation technique for static images based on multi-level semantic fusion. Our method utilizes user-guidance information both inside and outside the target object to segment it from the static image, making it applicable to both 2D and 3D sensor data. The proposed method introduces a cross-stage feature aggregation module, enabling the effective propagation of multi-scale features from previous stages to the current stage. This mechanism prevents the loss of semantic information caused by multiple upsampling and downsampling of the network, allowing the current stage to make better use of semantic information from the previous stage. Additionally, we incorporate a feature channel attention mechanism to address the issue of rough network segmentation edges. This mechanism captures richer feature details from the feature channel level, leading to finer segmentation edges. In the experimental evaluation conducted on the PASCAL Visual Object Classes (VOC) 2012 dataset, our proposed interactive image segmentation method based on multi-level semantic fusion demonstrates an intersection over union (IOU) accuracy approximately 2.1% higher than the currently popular interactive image segmentation method in static images. The comparative analysis highlights the improved performance and effectiveness of our method. Furthermore, our method exhibits potential applications in various fields, including medical imaging and robotics. Its compatibility with other machine learning methods for visual semantic analysis allows for integration into existing workflows. These aspects emphasize the significance of our contributions in advancing interactive image-segmentation techniques and their practical utility in real-world applications.

## 1. Introduction

Interactive image segmentation (IIS) is a technique that utilizes user-interaction input to isolate target objects and plays a significant role in the field of image and video editing [1] and medical diagnosis [2]. IIS provides higher accuracy than automatic segmentation, especially in fields like image and video editing or medical diagnosis, where high-precision segmentation results are necessary or personal safety is at risk. Due to the lower segmentation accuracy of the automatic segmentation model and the lack of human audit control, IIS is a necessity in such fields. Researchers have conducted extensive research in this field, with recent work by Papadopoulos et al. [3] demonstrating a more efficient method of obtaining bounding boxes using extremal clicks. The study demonstrated that extreme clicking, which took an average of 7.2 s, produced high-quality bounding boxes comparable to those obtained by traditional methods that require 34.5 s for object detection. These extremum points belong to the four extremum points of the upper, lower, left, and right sides of the object, containing four points located on the object boundary from which the bounding box can be easily obtained. The deep extreme cut (DEXTR) method [4] leverages these extreme points to generate an attention Gaussian map, which is then used as an additional channel to fuse with the original red/green/blue (RGB) image and input it into the segmentation network. The network learns to map the four extreme points of the input into a mask of the target object. Additionally, in order to concentrate on the objects of interest, the input is cropped using a bounding box created from boundary point annotations. Moreover, to encompass context in the outcomes, the tight bounding boxes are slightly expanded by a few pixels. After the preprocessing step of extreme clicks, the input consists of RGB cropped images containing objects plus their extreme points. The feature backpropagating refinement scheme (F-BRS) method [5,6] proposes an improved scheme of feature backpropagation that reframes the parameter optimization problem, which runs forward and backward passes through a part of the network, namely the last few layers. The method introduces a set of auxiliary parameters for optimization, which can be optimized without computing and passing backwards through the entire network, making it more efficient.

The terms “internal” and “external” guidance refer to the user-generated guidance points located inside and outside the object during the interaction process. The foreground point, which is an internal guidance point, helps the model learn the internal characteristics of the object and benefits the segmentation process. On the other hand, the background points, which are external guidance points, assist the model in distinguishing between the foreground and background and thus enhance the segmentation performance of the model. The application of semantic and instance segmentation has seen significant advancements in various domains, such as general scenes, image editing, and medical diagnosis. However, creating the pixel-level training data necessary for building successful segmentation models can be time consuming, laborious, and expensive. Interactive segmentation offers an attractive and effective way to reduce the annotation workload by allowing human annotators to quickly extract objects of interest with some user input, such as bounding boxes or clicks. Recently, Maninis et al. [4] proposed using the extreme points for IIS objects, including the leftmost, rightmost, top, and bottom pixel points, which enables fast interactive annotation and high-quality segmentation. Additionally, Zhang et al. [7] explored an inner–outer guidance approach, which involves one foreground click and four background clicks for segmentation. Specifically, the method uses an inner point near the center of the object and two outer points at symmetrical corner positions of a tight bounding box around the target object. The approach attains state-of-the-art results on various popular benchmarks and showcases its generalization ability in diverse domains, including street scenes, aerial images, and medical images. However, the click process can be further optimized to reduce annotation time and effort.

The proposed method incorporates several key components, including the selection of inner and outer guide points and the utilization of channel attention mechanisms. These components were inspired by prior research in the field. In terms of the inner and outer guide points selection, previous studies have demonstrated their effectiveness in various computer vision tasks. Maninis et al. [8] proposed a one-shot video object segmentation approach that leverages inner and outer boundary awareness for accurate segmentation. Similarly, Zheng et al. [9] introduced the concept of inner and outer frames to rethink semantic segmentation from a sequence-to-sequence perspective. These works highlight the importance of considering both inner and outer cues in achieving precise and robust segmentation results. Regarding channel attention mechanisms, researchers have explored different strategies to enhance the modeling capabilities of convolutional neural networks. Zhu et al. [10] introduced squeeze-and-excitation networks (SENet), which adaptively recalibrate channel-wise feature responses to capture informative features. Another approach, proposed by Woo et al. [11], is the convolutional block attention module (CBAM), which incorporates both spatial and channel attention mechanisms. Additionally, Zhang et al. [12] presented self-attention generative adversarial networks (SAGANs), which employ self-attention mechanisms to model long-range dependencies in images.

The field of machine learning-based applications, such as object detection, scene segmentation, and salient object detection, relies heavily on understanding and analyzing 2D/3D sensor data. Interactive object segmentation is a crucial task in image editing and medical diagnosis, requiring the accurate separation of target objects from their backgrounds based on user annotation information [13,14,15]. However, existing methods struggle to effectively leverage user guidance information to guide segmentation models. This paper presents a novel interactive image segmentation technique for static images based on multi-level semantic fusion. The proposed method aims to utilize user guidance information both inside and outside the target object to achieve precise segmentation. It can be applied to both 2D and 3D sensor data, making it versatile for various applications. The main contributions of the proposed method can be summarized as follows:

(1) Multi-level semantic fusion: The method incorporates a cross-stage feature aggregation module that facilitates the effective propagation of multi-scale features from previous stages to the current stage. This module mitigates the loss of semantic information caused by multiple upsampling and downsampling operations, enabling the current stage to make more complete use of the semantic information from the previous stage. This multi-level fusion approach enhances the overall segmentation accuracy. (2) Fine segmentation edges: To address the issue of rough network segmentation edges, the method includes a feature channel attention mechanism. This mechanism captures richer feature details at the channel level, resulting in finer segmentation edges. By emphasizing important features and suppressing less relevant ones, the proposed attention mechanism improves the overall quality of the segmented object boundaries.

## 2. Problem Description

In natural image segmentation tasks, achieving the fine segmentation of objects of different scales is crucial since objects in natural images vary greatly in scale. The feature pyramid network (FPN) [16], a fundamental component used for detecting objects of different scales, employs a horizontally connected top–down architecture to construct high-level semantic feature maps at all levels. This general-purpose feature extractor performs well in natural image-segmentation tasks. Meanwhile, the pyramid scene parsing network (PSPNet) [17] utilizes a feature pyramid pooling module to fuse features from multiple scales, effectively utilizing global contextual information. However, the repeated up-and-down sampling in the multi-level network can result in the loss of information, which causes blurred edges in the segmentation results. Therefore, reducing the loss of information during repeated up-and-down sampling in multi-level networks is a valuable research direction. In addition, previous IIS methods based on extreme points such as DEXTR [4] do not introduce background channels as segmentation information, which makes the model fail to focus on more background information for auxiliary segmentation. To address this, interaction information can effectively capture the user’s real intention and improve the precision and accuracy of the segmentation results.

Increasing the network depth can significantly improve the model learning quality. Batch normalization stabilizes the learning process in deep networks by adjusting input distributions at each layer, resulting in smoother optimization. However, with the increased depth of the segmentation network, the better integration of spatial and channel information at each layer to construct informative features and masking feature channels that are irrelevant to the segmentation task have become a valuable research area.

Based on the specific issues described above, this paper proposes an interactive image-segmentation method that utilizes multi-level semantic fusion. This method employs a cross-stage feature fusion strategy to transfer multi-scale feature information from the previous stage to the current stage. Thus, the current stage can make full use of prior information to extract more discriminative representations. The method introduces channel attention to perceive the relationship between different feature channels and assign corresponding weights to them. This increases the ability of the model to obtain more feature information. After training, the method can increase the weights of the features that improve the final segmentation accuracy according to the global information and reduce the weights of the features that do not improve the final segmentation accuracy.

## 3. Method

### 3.1. An Interactive Image-Segmentation Method Based on Multi-Level Semantic Fusion

**Figure 1:**
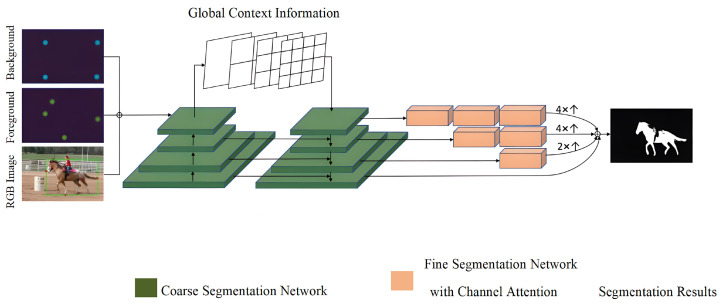
The overall framework of the interactive image-segmentation method based on multi-level semantic fusion.

The current paper builds upon the DeepLabv3+ network [18] and employs a coarse-to-fine structure [19] for segmentation, in which two subnets are used to segment the images from coarse to fine levels. Similar to inside–outside guidance (IOG) [7], the first subnet, CoarseNet, utilizes a feature pyramid network, which merges low-level semantic features with deeper semantic features through lateral connections. Additionally, a feature pyramid scene analysis network is incorporated at the deepest level to extract diverse features. Moreover, a cross-stage feature fusion strategy is employed in CoarseNet to enhance the discriminative power of the network. FineNet, the second subnet, receives the coarse-grained predictions from CoarseNet and enhances the segmentation by restoring the missing boundary details. This is accomplished using a multi-scale fusion architecture that integrates information from various levels in CoarseNet through upsampling and cascading operations. To ensure better accuracy and efficiency, FineNet applies more convolutional blocks to deeper features. Channel attention is introduced in FineNet to assign appropriate weights to feature channels, which enhances the model’s ability to capture feature information. Specifically, a feature weight redistribution method is designed to increase the weight of the features that improve the final segmentation accuracy and decrease the weight of the features that do not. Figure 1 depicts the overall framework of the network structure.

The CoarseNet structure is a crucial component of our proposed method for interactive image segmentation. It is designed to capture multi-scale features and establish a strong initial segmentation. The structure consists of several convolutional layers and pooling operations arranged in a cascaded manner. This cascaded architecture allows for the extraction of features at different scales and levels of abstraction. By incorporating skip connections, the CoarseNet can effectively combine low-level and high-level features to enhance the representation capability of the network. This integration of multi-scale features helps to capture both local details and global context, leading to improved segmentation performance. The FineNet structure plays a key role in refining the initial segmentation obtained from the CoarseNet. It aims to further enhance the segmentation accuracy and generate precise object boundaries. The FineNet structure consists of additional convolutional layers and upsampling operations. It takes the coarse segmentation map from the CoarseNet as input and performs further feature extraction and refinement. By upsampling the coarse segmentation map, the FineNet can generate a high-resolution segmentation output that preserves fine details and produces more accurate object boundaries. This refinement process is essential for achieving precise and visually appealing segmentations.

The present paper defines internal and external guidance points for an interactive image segmentation method. The former comprises four points: the object’s top, bottom, left and right extreme points that the user selects, forming the internal bootstrap information. The latter refers to the four vertices of the minimum circumscribed rectangular frame of the object that the system automatically deduces from the four internal guide points. To generate Gaussian heat maps centered on each guide point, users click on the representation, and two separate heat maps for inner and outer guide points are created using the same representation as DEXTR [4]. The generated heat maps are then connected to the RGB input image, producing the 5-channel network input. To ensure better coverage of the background, the object’s bounding box is framed by a few pixels before cropping out the object. This improves the network’s focus on the target object. Compared to click-based and bounding box-based methods, the proposed interactive method offers two advantages: (1) it is easy to expand, and the four extreme points marked by the user are encoded as foreground channels, while the four background points obtained by automatic reasoning are encoded as background channels, allowing further refinement of the segmentation results through additional user click annotations, and (2) it contains more information, implying more prior knowledge about the segmented object, such as its rough size and foreground and background position information.

#### 3.1.1. Cross-Stage Feature Aggregation Module

**Figure 2:**
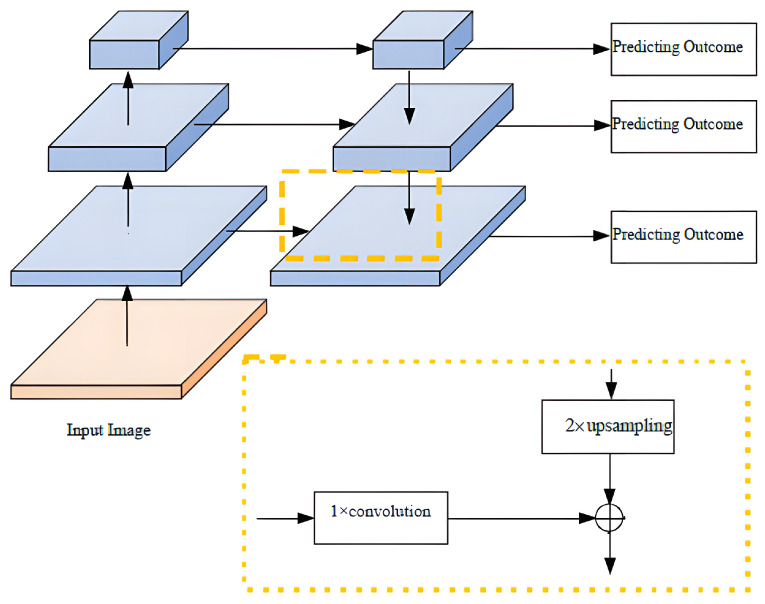
Module diagram of lateral connections and top–down pathways.

CoarseNet performs across-stage feature aggregation using a structure similar to FPN [16], which is a popular method for image segmentation. FPN consists of downsampling and upsampling modules and residual connections that improve segmentation accuracy. CoarseNet adopts a ResNet-based network structure and further enhances it with deep supervision information. It uses a fully convolutional structure to calculate feature maps of multiple scales at different levels, allowing it to handle images of various sizes. The pyramid structure of the network includes routes from the top to the bottom and vice versa, and the fusion of horizontal features. The bottom-up route is formed by feature transformation in several dimensions, with multiple layers generating outputs of the same size in the same stage. The method in this paper determines the corresponding level for each stage of the feature pyramid. The bottom-level results of each stage are used as a reference set for feature transformation, enriching them to create a pyramid. This design is based on the fact that the most abundant information is available at the bottom of each stage. Figure 2 shows the top–down paths and lateral connections. The structure enables the up-sampling of features from the upper pyramid, which are relatively coarse-grained but contain strong semantic information due to being sampled less frequently. Horizontal connections enhance feature information by fusing it from both the bottom–up and top–down routes, with features of the same dimensions. While the bottom–up feature transformation has lower-level semantic information, its subsampling times are relatively low due to the more precise location features.

**Figure 3:**
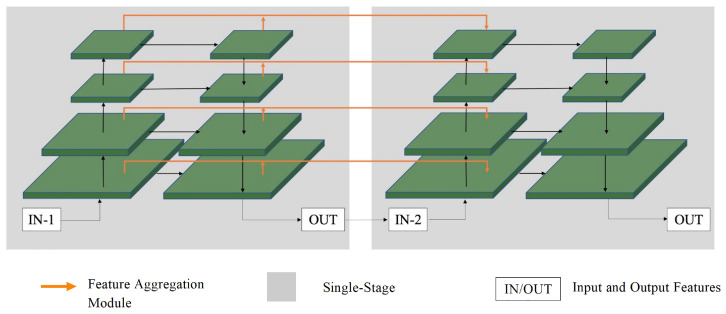
Framework diagram of cross-stage feature aggregation module.

The multi-level network contains numerous layers, which can lead to information loss due to downsampling and upsampling at each level. To address this issue, cross-stage feature aggregation methods are employed to extract more discriminative representations by utilizing prior information from the current stage. Figure 3 illustrates the introduction of two separate information flows from the downsampling and upsampling units of the previous stage to the downsampling unit of the current stage for each layer.

**Figure 4:**
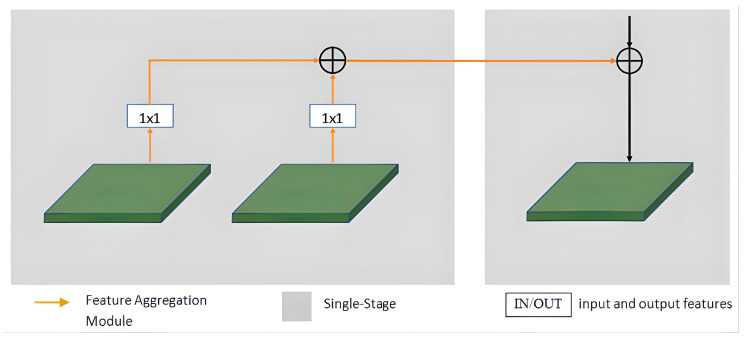
Framework diagram of cross-stage feature aggregation at a specific scale.

To address the issue of information loss that can arise due to the presence of numerous layers in a multi-level network, cross-stage feature aggregation methods are utilized. This helps each stage extract more discriminative representations by leveraging prior information. In Figure 3, it is demonstrated that for each layer, two distinct information flows are incorporated from the previous stage’s downsampling unit and upsampling unit to the current stage’s downsampling unit. To enhance this process, a 1×1 convolution operation is added to each information flow, as illustrated in Figure 4. The fusion feature information of the previous stage is then added after the current stage undergoes a downsampling operation. This design enables the current stage to fully utilize the prior information and extract more discriminative representations.

The application of cross-stage feature aggregation design in multi-level networks can effectively fuse contextual feature information and mitigate information loss caused by repeated up-and-down sampling. Moreover, aggregating features at different stages strengthens the information flow, easing the difficulty of training and improving high spatial resolution and multi-level supervision. Specifically, the coarse segmentation network follows a design similar to the feature pyramid network, ensuring better segmentation accuracy. To fully utilize multi-level information, a feature information aggregation module is designed. This module enables the later stages of the model to obtain feature information from the previous stage. As a result, the feature information between multiple stages is enriched, leading to improved segmentation accuracy and more discriminative representation across scales.

#### 3.1.2. Channel Attention

**Figure 5:**
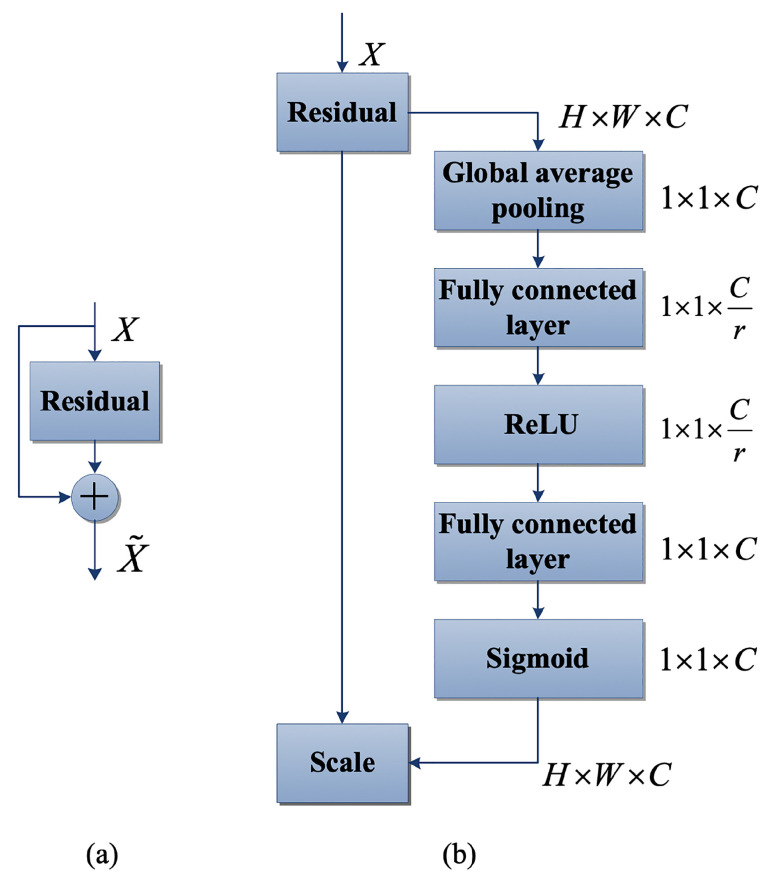
Module diagram: (**a**) the original residual module, and (**b**) SE-ResNet module.

Channel attention is a mechanism that assigns weights to different feature channels based on their perceived importance, thereby enhancing the model’s ability to extract feature information. In this paper, we design a lightweight channel attention mechanism based on the approach proposed by Hu et al. [20] as illustrated in Figure 5. Additionally, we introduce a method for redistributing feature weights after training, which increases the weight of features that improve final segmentation accuracy according to global information, and reduces the weight of features that do not improve it as shown in Equation (1). This weight redistribution can be achieved by designing modules of the squeeze-and-excitation (SE) structure in convolution. First, feature U is compressed to output channel feature information of size H×W, which reflects the global information and enables lower layers to utilize it. Then, an incentive operation converts the relationship between feature channels into different weight values for each channel, and feature U is reweighted to generate the output of the SE block, which is fed to other layers in the network. The SE network comprises superimposed SE blocks that can replace initial blocks in different layers, and each block plays a different role in meeting network requirements. In the upper network, SE blocks excite features in a class-agnostic manner to improve the sharing of lower-level representation information, while in the lower network, they become more specialized to reflect different types of inputs in specific classes. Consequently, the SE block’s role in redistributing feature weights is continuously enhanced in the network. Compared to designing new convolutional neural network (CNN) frameworks, the design of the SE module is simpler and can be easily integrated with existing state-of-the-art methods. Furthermore, its functional modules can be enhanced by quickly replacing SE modules. Additionally, the SE block’s lightweight design only slightly increases the complexity and operation cost.

A compression and excitation block is a computational unit that can be constructed for a specified number of transformations. The specific conversion method is depicted in Equation (1):

(1) $$F_{tr}:X\to U,\;\;X\in R^{H^{{}^{\prime }}\times W^{{}^{\prime }}\times C^{{}^{\prime }}},\;\;U\in R^{H\times W\times C}$$

Let $F_{tr}$ be the convolution operator, and let $V=[v_{1},v_{2},\ldots ,v_{C}]$ denote the set of trainable filter kernels, where $v_{C}$ is the parameter of the C-th filter. The output of $F_{tr}$, denoted by $U=[u_{1},u_{2},\ldots ,u_{C}]$, is defined in Equation (2):

(2) $$u_{c}=v_{c}*X=\sum_{s=1}^{C^{{}^{\prime }}}v_{c}^{s}*X^{s}$$

The equation presented in the previous paragraph shows the convolution operation denoted by the symbol ∗. Additionally, let $v_{c}=\left[v_{c}^{1},v_{c}^{2},\ldots ,v_{c}^{C^{{}^{\prime }}}\right]$, $X=[x^{1},x^{2},\ldots ,x^{C^{{}^{\prime }}}]$ be defined as the 2D spatial kernel and a single channel of $v_{C}$, respectively. Furthermore, $v_{c}^{s}$ can be utilized in the corresponding channel of X. As the output is the sum of all channels, the relationship between channels is encoded into $v_{c}$. Moreover, this relationship overlaps with the spatial correlation obtained by the filter, which emphasizes the need for the attention module to enhance its feature-acquisition capabilities. This feature acquisition ability further assists the network with suppressing invalid transformations at later stages.

Compression is utilized to encode global contextual features and address the problem of using the correlation between feature channels. It is important to consider the relevant information of all channels in the input and output. Since all trainable filters are trained using a local receptive domain, each unit in the output map U cannot utilize feature information outside of this domain. This problem may be particularly severe in networks with small receptive area widths. To address this, global features can be compressed into channel features. This is achieved by applying global average pooling to generate statistical information of the channel dimension feature. Specifically, compressing U into a dimension such as H×W generates a statistical result $z\in R^{C}$. The c-th factor of this result is calculated using Equation (3):

(3) $$z_{c}=F_{sq}\left(u_{c}\right)=\frac{1}{H\times W}\sum_{i=1}^{H}\sum_{j=1}^{W}u_{c}\left(i,j\right)$$

The collection of local descriptors in U contains statistics that are globally available on the image. Such feature information is commonly used in feature-engineering operations. Although the average pooling operation used here is the simplest to compute, more complex aggregation strategies can be used if necessary. The purpose of achieving self-adaptation and resetting weights can be accomplished through the use of incentives. To fully capture the dependency of feature channels, it is necessary to use the feature information aggregated after compression, which requires meeting two conditions. Firstly, it should possess good flexibility to learn non-linear correlations between channels. Secondly, it should have the ability to learn non-mutually exclusive relations, which enables it to activate multiple feature channels rather than just one. To meet these criteria, a simple gating mechanism with sigmoid activation is chosen, as illustrated in Equation (4):

(4) $$s=F_{ex}\left(z,W\right)=\sigma \left(g\left(z,W\right)\right)=\sigma \left(W_{2}\delta \left(W_{1}z\right)\right)s$$

where δ represents the rectified linear unit (ReLU) function [21], $W_{1}\in R^{\frac{C}{r}\times C}$, $W_{2}\in R^{\frac{C}{r}\times C}$. Finally, the transformed output U is rescaled by using an activation function to obtain the final output of the block as shown in Equation (5):

(5) $${\tilde{x}}_{c}=F_{scale}\left(u_{c},s_{c}\right)=s_{c}\cdot u_{c}$$

where $\tilde{X}=\left[{\tilde{x}}_{1},{\tilde{x}}_{2},\ldots ,{\tilde{x}}_{c}\right]$, $F_{scale}\left(u_{c},s_{c}\right)$ refers to the channel multiplication between feature map $u_{c}\in R^{H\times W}$ and scalar $s_{c}$. The incorporation of a channel attention mechanism in the network facilitates the effective utilization of the dependency information between feature channels. It enables the full utilization of the feature channels that are helpful in achieving segmentation while suppressing the feature channels that are not useful in producing segmentation results.

The loss function employed to train the entire network structure in our proposed method for interactive image segmentation is a combination of two components: the binary cross-entropy loss and the boundary loss. The binary cross-entropy loss is widely used for binary classification tasks and is applied to each pixel of the segmented output. It measures the dissimilarity between the predicted segmentation probabilities and the ground truth annotations. The binary cross-entropy loss encourages the network to produce segmentation predictions that align with the ground truth labels, promoting accurate object boundary separation and background suppression. The boundary loss component focuses on capturing the fine details and precise object boundaries. It is designed to penalize incorrect boundary predictions and encourage the network to generate sharp and accurate object contours. The boundary loss measures the difference between the predicted boundary map and the ground truth boundary map. By incorporating the boundary loss, the network is trained to capture the intricate details of object boundaries, resulting in visually appealing segmentations. The overall loss function is computed as a weighted sum of the binary cross-entropy loss and the boundary loss. The weights are adjusted to balance the importance of each loss component in the training process. By jointly optimizing these components, the network learns to produce accurate segmentations while preserving fine object details and boundaries. By using this combination of the binary cross-entropy loss and the boundary loss, our network is trained to produce accurate segmentations with well-defined boundaries, resulting in improved performance in the interactive image segmentation task.

The choice of the specific loss function used in our proposed method for interactive image segmentation, which combines binary cross-entropy loss and boundary loss, is driven by the objectives and requirements of the task. By combining the binary cross-entropy loss and the boundary loss in our proposed loss function, we leverage the strengths of both components to achieve the overall objectives of accurate object segmentation and boundary refinement. The binary cross-entropy loss ensures the accurate separation of the object from the background, while the boundary loss enhances the network’s ability to capture detailed object contours. The rationale behind the choice of this loss function is to strike a balance between accurate object segmentation and precise boundary delineation, ultimately improving the overall performance of interactive image segmentation.

## 4. Experimental Results

A comparison test and ablation experiment were conducted on the proposed network using the public image segmentation dataset to evaluate its effectiveness. This paper presents the experimental results and ablation test outcomes, comparing them with current advanced methods. Simultaneously, the experimental results are analyzed.

### 4.1. Datasets and Evaluation Criteria

The training dataset employed in this study is based on the PASCAL public dataset (website accessed on 6 February 2023: http://host.robots.ox.ac.uk/pascal/VOC/), which was mentioned in [22]. It consists of twenty different object classes, including animal classes, such as bird, cat, cow, dog, horse, and sheep; vehicle classes, including airplane, bicycle, boat, bus, car, motorcycle, and train; indoor object classes, such as bottle, chair, dining table, potted plant, sofa, TV, and monitor as well as people. The training and validation datasets contain 11,530 images, encompassing 27,450 region of interest (ROI) annotated objects and 6929 segmentation annotations. Table 1 displays the statistics of the segmented image set. The model evaluation adheres to the standards specified in the PASCAL public dataset.

The evaluation metric primarily used for the interactive image segmentation task is IOU, which is a widely adopted measure for image segmentation. IOU is calculated as the ratio of the intersection area between the predicted segmentation map and the ground truth segmentation to the union area between the predicted segmentation map and the ground truth segmentation as depicted in Equation (6):

(6) $$\mathrm{IOU}=J\left(A,B\right)=\frac{\left|A\cap B\right|}{\left|A\cup B\right|}$$

where *A* and *B* in the equation represent the segmentation ground truth and the predicted segmentation map, respectively. The IOU ranges between 0 and 1, and is a commonly used metric in image segmentation. Mean IOU is another widely used evaluation metric, defined as the average of IOU values across multiple object classes. It is used to demonstrate the overall accuracy of segmentation algorithms.

### 4.2. Lab Environment

This method is implemented using the PyTorch deep learning framework, and the experiment is conducted on two NVIDIA GeForce RTX 1080Ti GPUs with 11G video memory. The network language is Python 3.6, and CUDA 9.0 is used for computing acceleration. The training process applies stochastic gradient descent (SGD) as an optimizer, with a weight decay of 0.0005 and a momentum of 0.9. The learning rate is set to $1\times 10^{-8}$, and a total of 100 epochs of training are performed on the PASCAL dataset [22].

### 4.3. Simulate Inner and Outer Guide Points

This paper employs segmentation ground truth to simulate user-generated clicks for training. The method extracts the top, bottom, leftmost, and rightmost four extreme point coordinates of the object to be segmented from the segmentation truth mask to simulate the user’s clicks for internal guide points. For external guide points, the method utilizes the four internal guide points to generate the smallest circumscribed rectangular frame around the object to be segmented. To realistically simulate the user’s input, the bounding rectangle is enlarged by 10 pixels. This approach more accurately mimics the user’s click process since it is difficult for the user to precisely click on the object’s four extreme points during the actual click process. The method relaxes a few extra pixels towards the periphery of the object to be segmented, ensuring that even if the click is inaccurate, the resulting circumscribed rectangle can still accurately surround the segmented object.

This study evaluates the robustness of the proposed method when users provide inaccurate clicks. During training, the model uses simulated user input instead of actual user input. However, in practical settings, users may not accurately mark the four extreme points of the object to be segmented, negatively impacting the model’s segmentation performance. Inconsistencies between training and testing can significantly affect the model’s performance in real-world scenarios. To evaluate the model’s robustness against inaccurate user clicks, robustness experiments were conducted, and the results are presented in Table 2. The table displays the IOU values of the segmented model after random perturbations at the input point. The experiments were performed by randomly disturbing a pixel within a specific range of the user’s original input to verify the model’s robustness. The statistical data in Table 2 indicates that when the disturbance varies from 5px to 25px, the impact on the final result ranges between 0.3% and 0.5%. The findings demonstrate that the proposed method exhibits good robustness.

**Figure 6:**
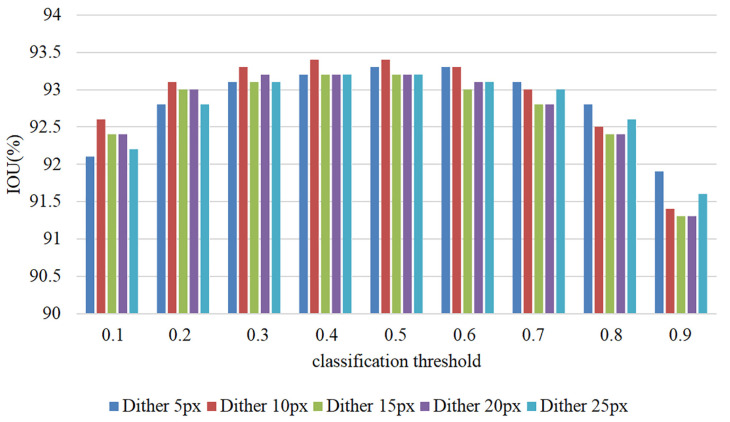
User click random perturbation experiment results.

Figure 6 demonstrates the intuitive impact of random perturbations on the robustness of the proposed method. The results suggest that a classification threshold of 0.5 results in the best robustness. Additionally, the method performs well in producing an IOU value when subjected to perturbations ranging from 5 px to 25 px.

### 4.4. Ablation Experiment

**Figure 7:**
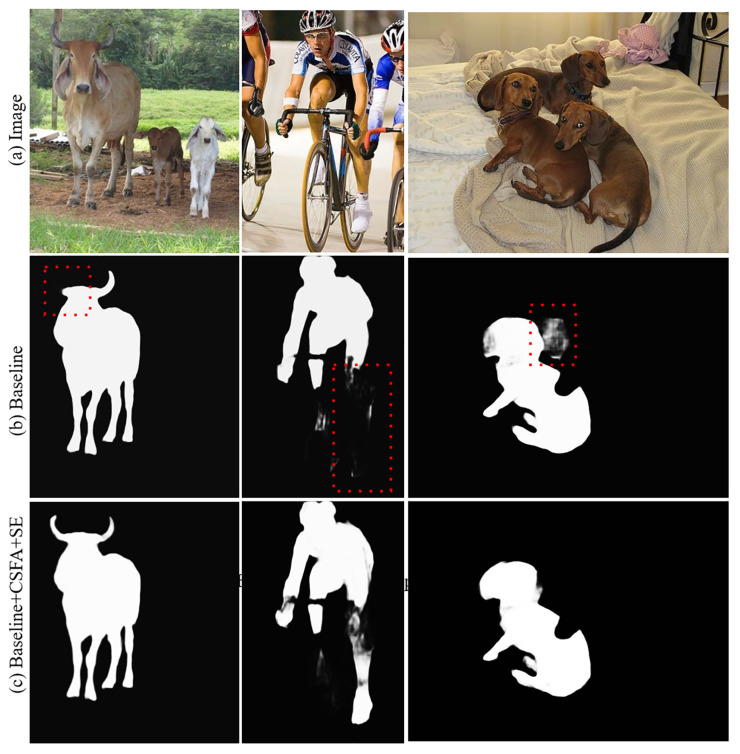
Visualization comparison of ablation experiments.

The paper conducted ablation experiments on the proposed cross-stage feature aggregation module and channel attention module to examine their effects on the model. The segmentation IOU values for each module are listed in Table 3 and illustrated in Figure 7. The letter “T” denotes the classification threshold, “CSFA” stands for cross-stage feature aggregation, and “SE” represents channel attention. The results demonstrate that both modules can enhance the final segmentation accuracy of the network. Furthermore, jointly applying the feature aggregation and channel attention modules produces better results than using either module alone, indicating that the modules have complementary effects. The cross-stage feature aggregation module can propagate multi-scale features from earlier stages to the current stage, allowing for the extraction of more discriminative representations using prior information. It facilitates information flow, training, and improves high spatial resolution and multi-level supervision. Moreover, it enriches feature information between multiple stages, which enhances the overall segmentation accuracy of the model and allows for the full use of contextual information and more discriminative representations across scales.

By incorporating the channel attention module in the network, the model gains the ability to discern the significance of distinct feature channels and allocate appropriate weights to them. This enhances the model’s ability to extract relevant feature information. Furthermore, after training, the proposed method is able to enhance the weight of features that contribute to better segmentation accuracy, based on the global information, while downgrading the weight of those that do not. It is noteworthy that the channel attention module has a lightweight design, adding only a negligible increase in the complexity and operation cost. Finally, the joint operation of both the cross-stage feature aggregation and channel attention modules results in an improvement of the network’s segmentation accuracy.

Based on Figure 7, it is evident that the basic network struggles to achieve complete segmentation of the target object without incorporating the cross-stage feature aggregation module and channel attention module. The limited capacity of the network to extract detailed features could be the reason behind its inability to fully utilize the rich feature information. However, upon integrating the cross-stage feature aggregation module and channel attention module, the network’s segmentation of the target object is smoother and more comprehensive. This is due to the enhanced ability to learn channel-dimensional features, which enables the model to leverage more information. Consequently, the addition of these two modules to the model results in improved segmentation performance compared to the basic approach.

**Figure 8:**
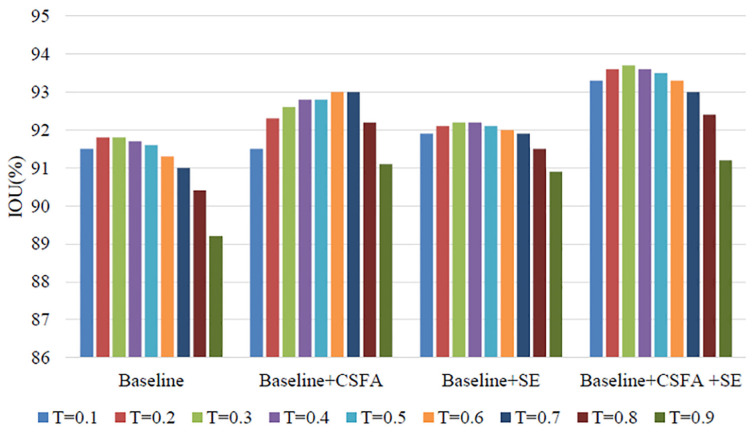
Comparison of IOU results in ablation experiments.

Based on the comparison chart of the results of the vertical comparative ablation experiment in Figure 8, it can be observed that using only the cross-stage feature aggregation module and the channel attention module does not result in the best performance of the model. This suggests that the potential of the model cannot be fully exploited by using these modules independently. However, when these two modules are used jointly, the segmentation accuracy of the model is significantly improved.

The segmentation accuracy of the model improved by approximately 1.9% after jointly applying two modules, indicating that the multi-scale fusion strategy proposed in this method enhances the flow of feature information in the model. Additionally, the channel attention mechanism improves the segmentation effect of the model by enabling it to learn in the feature channel dimension.

## 5. Discussion

**Figure 9:**
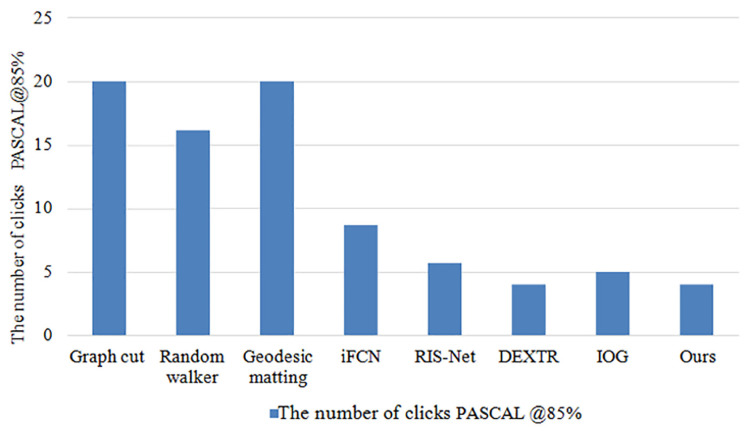
Comparison charts of the results of the number of clicks in the comparison experiment.

**Figure 10:**
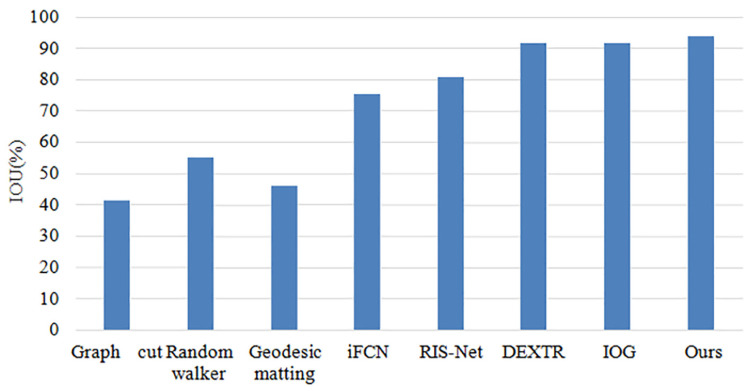
Comparison of IOU results in comparison experiments.

The comparison results between the methods used in this paper and several currently popular methods on public datasets are shown in Table 4, and Figure 9 and Figure 10. The results indicate that the method proposed in this paper achieves the highest IOU and requires the least number of interactions. This improvement can be attributed to the combination of the cross-stage feature aggregation module and the channel attention module.

The cross-stage feature aggregation module enhances the feature information flow of the current stage network, facilitating the transfer of context information and enriching the feature information of the current stage. On the other hand, the channel attention module allows the network to perceive the inter-channel relationship, and leverages it to improve the network’s ability to select relevant features, thus effectively utilizing valuable feature information while disregarding useless ones.

This attention mechanism is lightweight and can enhance the network’s ability to select relevant features and redistribute weights without adding significant computational cost. The mechanism allows the network to re-learn the relationships between channels, improving the learning ability of relevant features for the final segmentation result while reducing the impact of irrelevant feature channels. Ultimately, this mechanism enhances the network’s ability to extract features.

Compared to other popular methods, the approach proposed in this paper requires only four clicks to reach 85% of the IOU value on the PASCAL public dataset. This finding demonstrates that this method is among the most efficient extreme point methods in terms of the number of clicks required to achieve the same level of accuracy. In addition, the segmentation IOU accuracy of various segmentation methods was evaluated using only four clicks on the PASCAL public dataset. The results indicate that the method proposed in this paper achieves an IOU value of 93.7%, which is 2.1% higher than the current popular method IOG.

**Figure 11:**
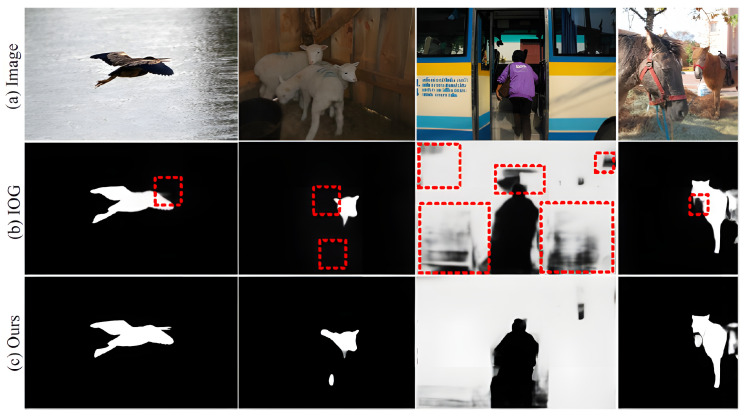
Visualization results of this method.

Based on Figure 3, Figure 4, Figure 5, Figure 6, Figure 7, Figure 8, Figure 9 and Figure 10, it can be concluded that the method proposed in this paper achieves highly accurate object segmentation while minimizing user interaction costs. Creating a training set with pixel-level annotations is a challenging task that requires users to manually perform pixel-level accurate segmentation of target objects in images. Achieving pixel-level annotation is difficult for humans, especially for irregular and rough edges, such as human hair or animal feathers. Therefore, reducing the cost of manual annotation is essential in the process of interactive segmentation. Figure 9 demonstrates that the method proposed in this paper requires the fewest user interaction clicks compared to other popular methods. Furthermore, the proposed method achieves a 2.1% increase in segmentation accuracy while reducing user interaction. These results validate the effectiveness of the proposed method in reducing manual interaction and improving segmentation accuracy. The results presented in Figure 11 demonstrate the high accuracy of the segmentation method proposed in this paper, which is capable of accurately segmenting objects of various scales and different parts of the same object. The resulting segmentation mask is both smooth and complete, effectively describing the original shape of the target object. These results highlight the effectiveness of the proposed cross-stage feature aggregation and channel attention modules, which improve the segmentation of multi-scale objects.

In our proposed method, we introduce an attention mechanism to enhance the modeling capabilities of our network. Specifically, we compare our attention module with the SE block used in SENet to demonstrate the effectiveness of our approach. The SE block, proposed by Hu et al. [20], focuses on recalibrating channel-wise feature responses in a network. It consists of a squeeze operation, which globally pools the input feature maps to capture channel-wise statistics, and an excitation operation, which learns a channel-wise excitation function to selectively emphasize informative features. The SE block has been widely adopted in various computer vision tasks and has shown improvements in performance. In our proposed method, we also incorporate a channel attention mechanism to assign appropriate weights to feature channels, allowing the network to focus on relevant and discriminative information. The method with the SE block in SENet achieved an IOU value of 90.2%, while ours achieved 93.7%, which is 3.5% higher.

By comparing our proposed attention mechanism with the SE block in SENet, we aim to demonstrate the effectiveness and superiority of our approach. (1) Capturing spatial and channel dependencies: while the SE block focuses on recalibrating channel-wise feature responses, our attention mechanism incorporates both spatial and channel dependencies. It allows our model to capture not only the importance of individual channels but also the spatial context within the feature maps. This comprehensive consideration of spatial and channel dependencies enables our method to better exploit the rich information present in the feature maps. (2) Integration with the network architecture: Our attention mechanism is seamlessly integrated into the network architecture, specifically in the FineNet subnet. It complements the existing structure and enhances its performance by providing refined feature representations. The attention module selectively highlights important features, enabling the network to focus on the most discriminative information for accurate segmentation. (3) Performance improvements: Through extensive experimental evaluations, we demonstrate that our attention mechanism yields superior performance compared to the SE block. The quantitative results show improved IOU, indicating the effectiveness of our approach in capturing and leveraging important features for the segmentation task. This study provides a comprehensive understanding of the benefits and advantages of our attention mechanism over the SE block. These improvements strengthen the credibility of our proposed method and highlight its potential for achieving state-of-the-art performance in interactive image segmentation tasks.

The qualitative attributes of this study lie in the following. (1) The visual quality of segmentation results: We compare the visual quality of segmentation results between our proposed method and related techniques. Through qualitative examples and visual comparisons, we demonstrate how our method achieves more accurate and precise object boundaries. The segmentations produced by our method exhibit smoother and more coherent object contours, resulting in visually appealing results. Additionally, we highlight scenarios where our method effectively handles challenging cases, such as complex backgrounds, object occlusions, or fine details, and produces superior segmentation quality compared to existing methods. (2) Robustness to challenging scenarios: We emphasize the robustness of our proposed method to challenging image conditions. We discuss how our approach handles difficult cases, including objects with low contrast, irregular shapes, and varying lighting conditions. By presenting qualitative examples, we demonstrate that our method maintains segmentation accuracy and robustness across a wide range of challenging scenarios. We show instances where our method successfully handles partial occlusions, object deformations, or variations in object appearance, showcasing its robustness compared to related techniques. (3) Handling of various object classes and shapes: We highlight the versatility of our proposed method in handling different object classes and shapes. Through qualitative comparisons, we discuss examples where our method effectively segments objects of various sizes, aspect ratios, and object categories. We showcase instances where our method accurately captures object boundaries, even for objects with complex shapes or instances with intricate boundaries. This demonstrates the ability of our method to handle diverse object characteristics and highlights its flexibility in different application domains.

The proposed method holds great potential for enhancing medical imaging applications, particularly in tasks such as tumor segmentation, organ delineation, and lesion detection. By leveraging multi-level semantic fusion and user guidance information, the method can accurately separate target objects from their backgrounds in medical images, leading to improved accuracy and reliability in diagnosis and treatment planning. This can aid in the better understanding and analysis of medical data, enabling clinicians to make informed decisions and improve patient care. Additionally, the compatibility of the proposed method with other machine learning techniques for visual semantic analysis allows for seamless integration into existing medical imaging workflows. Interactive image segmentation plays a crucial role in perception and scene understanding for autonomous vehicles. The proposed method can be effectively utilized to segment and separate objects of interest from the surrounding environment, enabling more precise and reliable object detection, tracking, and recognition. By incorporating multi-level semantic fusion and user guidance information, the method can enhance the accuracy and robustness of object segmentation in complex driving scenarios. This, in turn, contributes to improved situational awareness, enabling autonomous vehicles to make more informed decisions and navigate safely. The compatibility of the proposed method with 3D sensor data further enhances its applicability in autonomous driving systems. In both medical imaging and autonomous vehicles, the benefits of the proposed method lie in its ability to leverage user guidance information, both inside and outside the target objects, and its multi-level semantic fusion capabilities. These aspects enable more accurate and reliable object segmentation, leading to improved performance in various real-world applications.

In our study, the exact time needed for segmentation can vary depending on several factors, including the complexity of the images, the computational resources available, and the specific segmentation algorithm employed. As we conducted our experiments on a high-performance computing platform, we were able to achieve efficient segmentation times. However, it is important to note that the computational time may differ based on the hardware and software configurations used.

It is important to note that while we discussed medical imaging and autonomous vehicles as examples, the proposed method’s applicability extends to other domains, where interactive image segmentation is crucial, such as robotics, augmented reality, and computer-aided design. Overall, the interactive image segmentation method based on multi-level semantic fusion has the potential to significantly enhance a wide range of real-world applications, enabling more accurate and reliable analysis of visual data and empowering advanced decision-making processes.

## 6. Conclusions

This paper proposes an end-to-end interactive method for segmenting target objects in natural images. The method addresses the issue of poor segmentation performance in large models with multi-scale object differences in natural image-segmentation scenarios. The method has three main innovations: First, a cross-stage feature aggregation module is introduced in the coarse segmentation network. This module enhances the flow of feature information from the previous stage and improves context information transmission. Second, a channel attention module is introduced in the fine segmentation network to model the correlation of feature channels, which enhances the network’s ability to select channel dimension features, resulting in improved segmentation accuracy. Third, this method achieves competitive IOU results on public datasets, and its segmentation accuracy is better than that of several current popular methods. Moreover, its IOU index surpasses that of the currently popular interactive image segmentation methods in static images by approximately 2.1%. The effectiveness of the interactive image segmentation method, which is based on multi-level semantic fusion and proposed in this paper, was validated through experiments. However, there are still some limitations, and further research is necessary in future work. The following are feasible research directions: (1) The proposed method exhibits insufficient segmentation accuracy for objects with a high occlusion ratio. When an object is obscured by other objects, it can hinder the user’s interaction process, and make it challenging for the network to completely segment the object. To reduce this error, additional training sets containing occluded objects should be included. (2) Along with improving the segmentation accuracy, the model’s segmentation speed is equally important, particularly in interactive segmentation. Faster segmentation can reduce waiting time, enabling users to complete more interactions within a fixed time, without wasting time waiting for the model to produce segmentation results. Hence, further optimization of the model’s running speed is necessary.

## Figures and Tables

**Table 1:** Statistical data of pictures in PASCAL segmentation image set.

Object Category	Number of Images in the Training Set	Number of Objects in the Training Set	Number of Images in the Validation Set	Number of Objects in the Validation Set
Airplane	88	108	90	110
Bike	65	94	79	103
Bird	105	137	103	140
Boat	78	124	72	108
Bottle	87	195	96	162
Bus	78	121	74	116
Car	128	209	127	249
Cat	131	154	119	132
Chair	148	303	123	245
Cow	64	152	71	132
Dining table	82	86	75	82
Dog	121	149	128	150
Horse	68	100	79	104
Motorcycle	81	101	76	103
People	442	868	445	865
Potted plant	82	151	85	171
Sheep	63	155	57	153
Sofa	93	103	90	106
Train	83	96	84	93
Television	84	101	74	98
Total	1464	3507	1449	3422

**Table 2:** Statistical data of user click random perturbation experiment.

Classification Threshold	Disturbance 5 px	Disturbance 10 px	Disturbance 15 px	Disturbance 20 px	Disturbance 25 px
0.1	92.1	92.6	92.4	92.4	92.2
0.2	92.8	93.1	93.0	93.0	92.8
0.3	93.1	93.3	93.1	93.2	93.1
0.4	93.2	93.4	93.2	93.2	93.2
0.5	**93.3**	**93.4**	**93.2**	**93.2**	**93.2**
0.6	93.3	93.3	93.0	93.1	93.1
0.7	93.1	93.0	92.8	92.8	93.0
0.8	92.8	92.5	92.4	92.4	92.6
0.9	91.9	91.4	91.3	91.3	91.6

**Table 3:** IOU (%) results of ablation experiments.

Network	T = 0.1	T = 0.2	T = 0.3	T = 0.4	T = 0.5	T = 0.6	T = 0.7	T = 0.8	T = 0.9
Baseline	91.5	91.8	91.8	91.7	91.6	91.3	91.0	91.4	89.2
Baseline + CSFA	91.5	92.3	92.6	92.8	92.8	93.0	93.0	92.2	91.1
Baseline + SE	91.9	92.1	92.2	92.2	92.1	92.0	91.9	91.5	90.9
Baseline + CSFA + SE	**93.3**	**93.6**	**93.7**	**93.6**	**93.5**	**93.3**	**93.0**	**92.4**	**91.2**

**Table 4:** The number of clicks and IOU (%) result.

Method	Number of Clicks (PASCAL@85%)	Number of Clicks (GrabCut@90%)	IOU (%)@4 Clicks on PASCAL	IOU (%)@4 Clicks on GrabCut
Graph cut [23] (2001)	>20	>20	41.1	59.3
Random walker [24] (2006)	16.1	15	55.1	56.9
Geodesic matting [25] (2007)	>20	>20	45.9	55.6
RIS-Net [26] (2017)	5.7	6	80.7	85.0
DEXTR [4] (2018)	4	4	91.5	94.4
IOG [7] (2020)	5	5	91.6	95.7
Ours	**4**	**4**	**93.7**	**96.5**

## Data Availability

All the datasets used in this manuscript are publicly available datasets (PASCAL public dataset accessed on 6 February 2023: http://host.robots.ox.ac.uk/pascal/VOC/, already in the public domain.
